# Supplementary material for: Mycobacterium tuberculosis Affects Protein and Lipid Content of Circulating Exosomes in Infected Patients Depending on Tuberculosis Disease State
Source: Biomedicines. 2022 Mar 27;10(4):783. doi: 10.3390/biomedicines10040783 (PMC9025801; doi:10.3390/biomedicines10040783)
Supplement: Supplementary file 1 [file biomedicines-10-00783-s001.zip › Figures S1-S6.pdf]

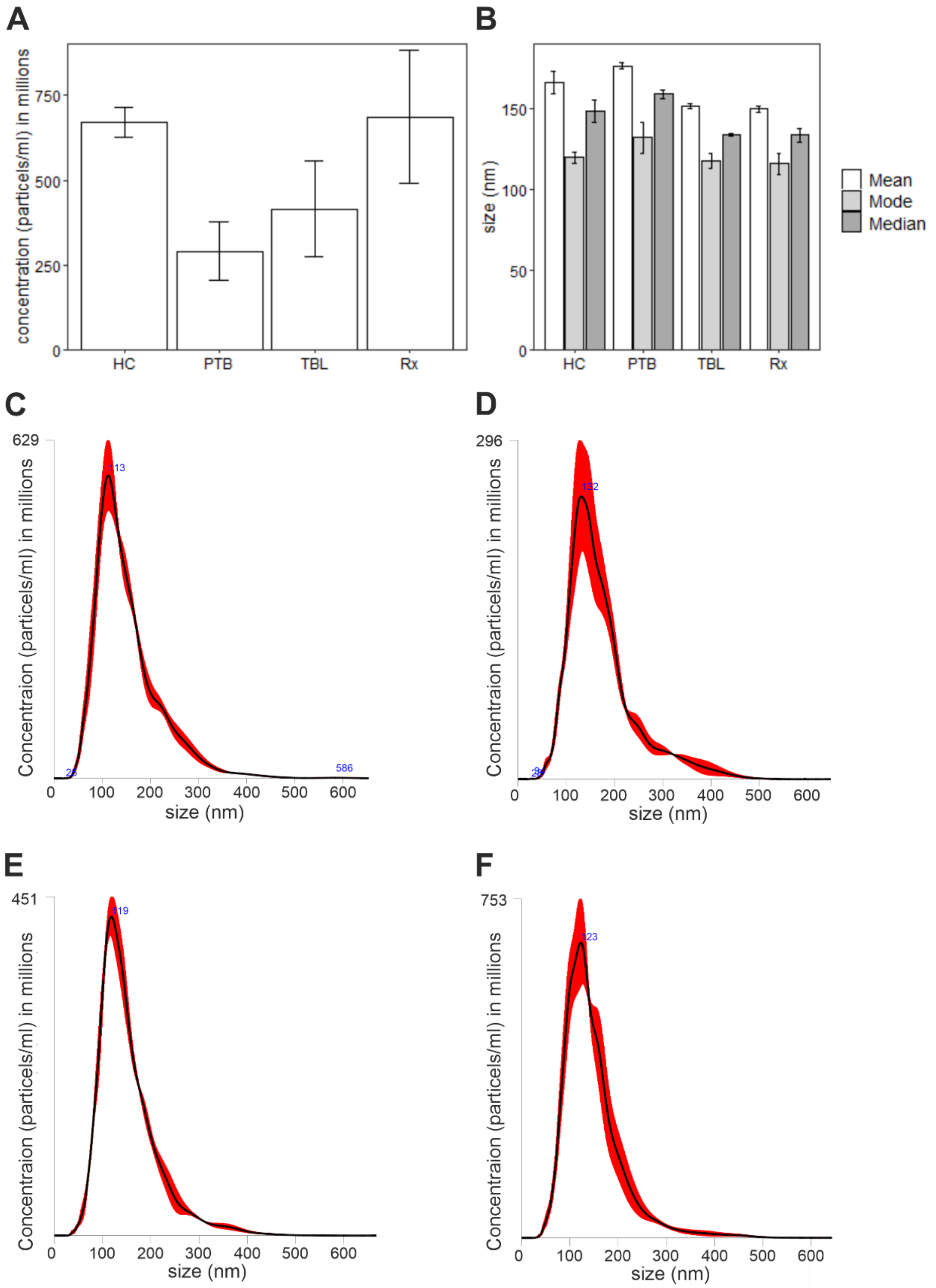


**Figure S1. Nanoparticle tracking analysis (NTA) of exosomes from blood plasma of healthy individuals.** (**A**) Concentration (particles/ml), (**B**) mean, mode and median value ± SEM of the diameter sizes (nm) measured by NTA. NTA distribution curves measured showing exosomes present in the blood plasma of HC (**C**), PTB (**D**), TBL (**E**) and Rx (**F**). The NTA setup for the analysis of size and concentration was a dilution factor of 1:500, camera level 9 and detection threshold of 4. Data included in the figure parts (C-F) represent the mean readings of three independent replicates. Red areas indicate standard error of the mean (SEM). HC - healthy controls, PTB - plumonary tuberculosis, TBL – tuberculous lymphadenitis, and Rx - PTB after anti-TB drug treatment, n=3 for all analyses.

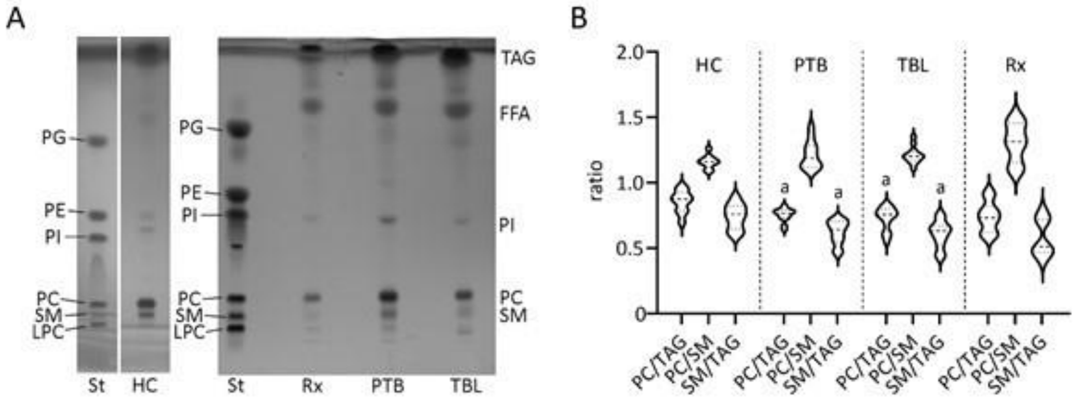


**Figure S2. HPTLC separation of lipid extracts from TB patients. A**) Extracted lipid-containing fractions from patients' blood plasma were separated on normal phase HPTLC glass plates (Merck KGaA, Darmstadt, Germany) using chloroform/ethanol/water/triethylamine (30:35:7:35, by vol.) as the mobile phase. For visualization of the separated lipids, the plates were dipped into primuline solution (Direct Yellow 59, Sigma-Aldrich, Taufkirchen, Germany; 50 mg/l in acetone/water (80:20, by vol.)), dried under room conditions for 15 min and illuminated with UV light (366 nm). One representative sample for each group is shown. (**B**) The density of the HPTLC spots of the SM, PC and TAG fractions of 9 HC, 13 PTB, 13 TBL and 6 Rx samples were analyzed by ImageJ (https://imagej.nhi.gov) and ratios were calculated. Data are shown as boxes and whiskers. The whiskers represent the highest and the lowest value, the solid line inside the boxes the mean. Please note that TAG and CE coelute under the used conditions and, thus, the TAG fraction also contains CE. CE – cholesteryl ester, LPC – lysophosphatidylcholine, PC – phosphatidylcholine, PE – phosphatidylethanolamine, PG – phosphatidylglycerol, PI – phosphatidylinositol, HC– healthy controls, PTB – pulmonary tuberculosis, Rx – PTB treated cases, S – lipid standard mixture, SM – sphingomyelin, TAG – triacylglycerol, TBL – tuberculous lymphadenitis.

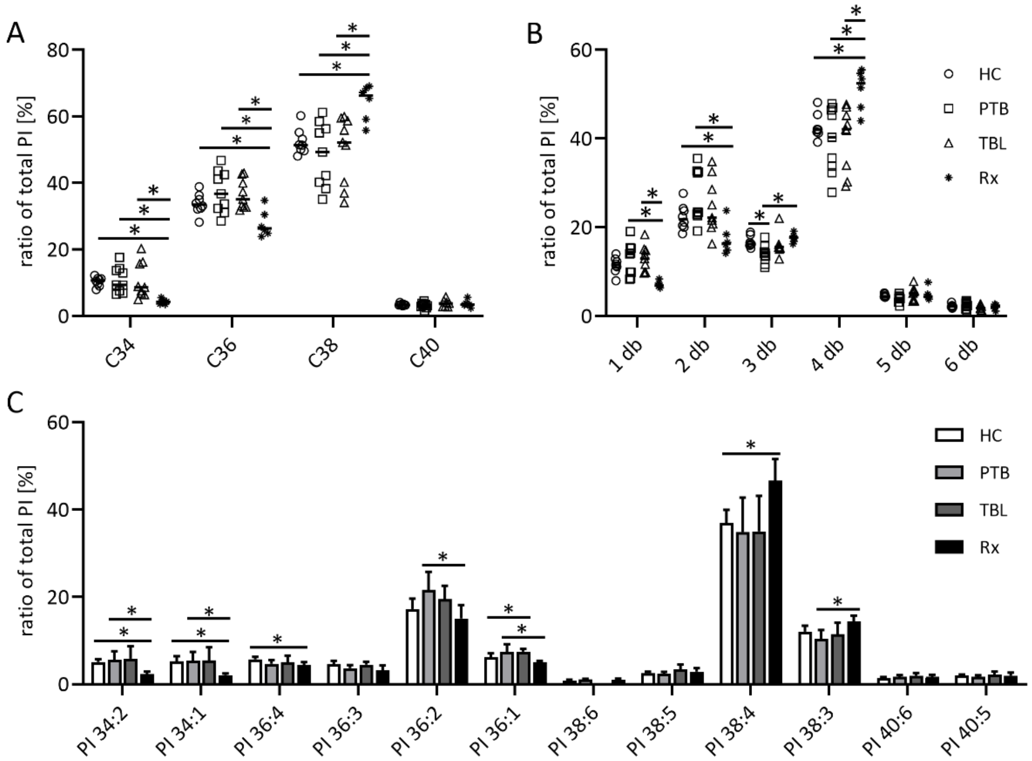


**Figure S3. Composition of the phosphatidylinositol (PI) fraction of exosomes from TB patients.** Organic extracts from patients with pulmonary toberculosis (PTB) and tuberculous lymphadenitis (TBL) as well as PTB-treated patients (Rx) were separated by high-performance thin layer chromatogarphy. PI-containing spots were automatically eluted with methanol and directly analyzed by ESI-IT MS. The sum of PI species with 34, 36, 38 and 40 C atoms (**A**) and the sum of PI species with a certain amount of double bonds (db) in their fatty acyl residues (**B**) as well as the relative amounts of single PI species (**C**) were calculated from the sum of the signal intensities of all PI. Data in (**A**) and (**B**) are depicted as dot plots to show all single values, data in (**C**) are depicted as bar graphs showing the mean and the positive standard deviation. Statistical significance was determined using the unpaired t test and the Holm-Sidak method to correct for multiple comparisons (alpha = 0.05). *$p < 0.05$, **$p < 0.01$, ***$p < 0.001$.

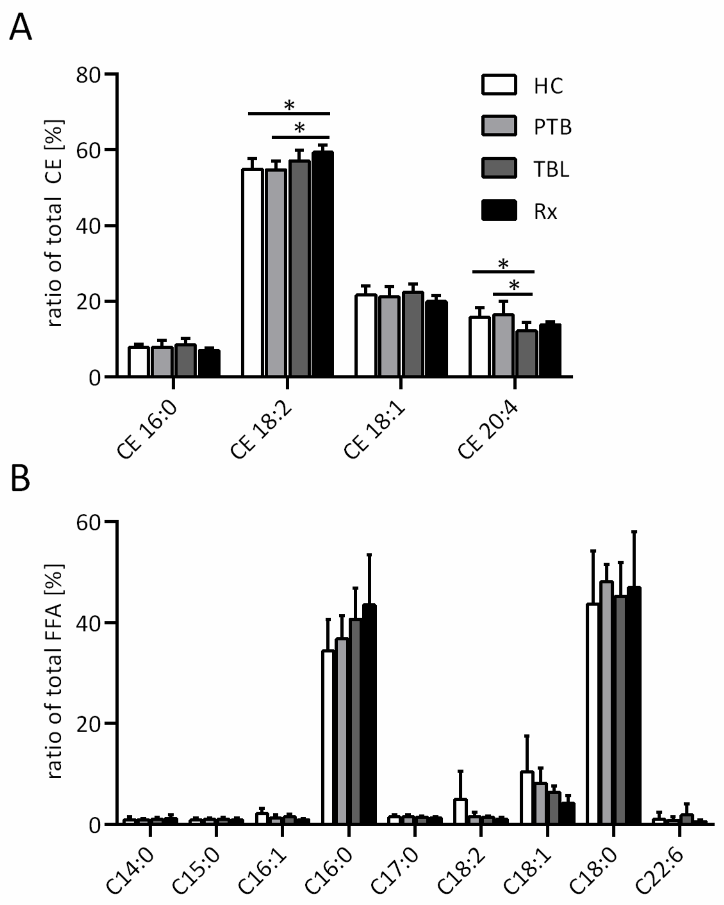


**Figure S4. Composition of the cholesteryl ester (CE) and the free fatty acid (FFA) fraction of exosomes from TB patients.** Organic extracts from patients with pulmonary toberculosis (PTB) and tuberculous lymphadenitis (TBL) as well as PTB patients after treatment (Rx) were separated by high-performance thin layer chromatography. CE- and FFA-containing spots were automatically eluted with methanol and directly analyzed by ESI-IT MS. Relative amounts of single (**A**) CE species and (**B**) FFA were calculated from the sum of the signal intensities of all CE or FFA, respectively. Data are depicted as bar graphs showing the mean and the positive standard deviation. Statistical significance was determined using the unpaired t test and the Holm-Sidak method to correct for multiple comparisons (alpha = 0.05). $*p < 0.05$.

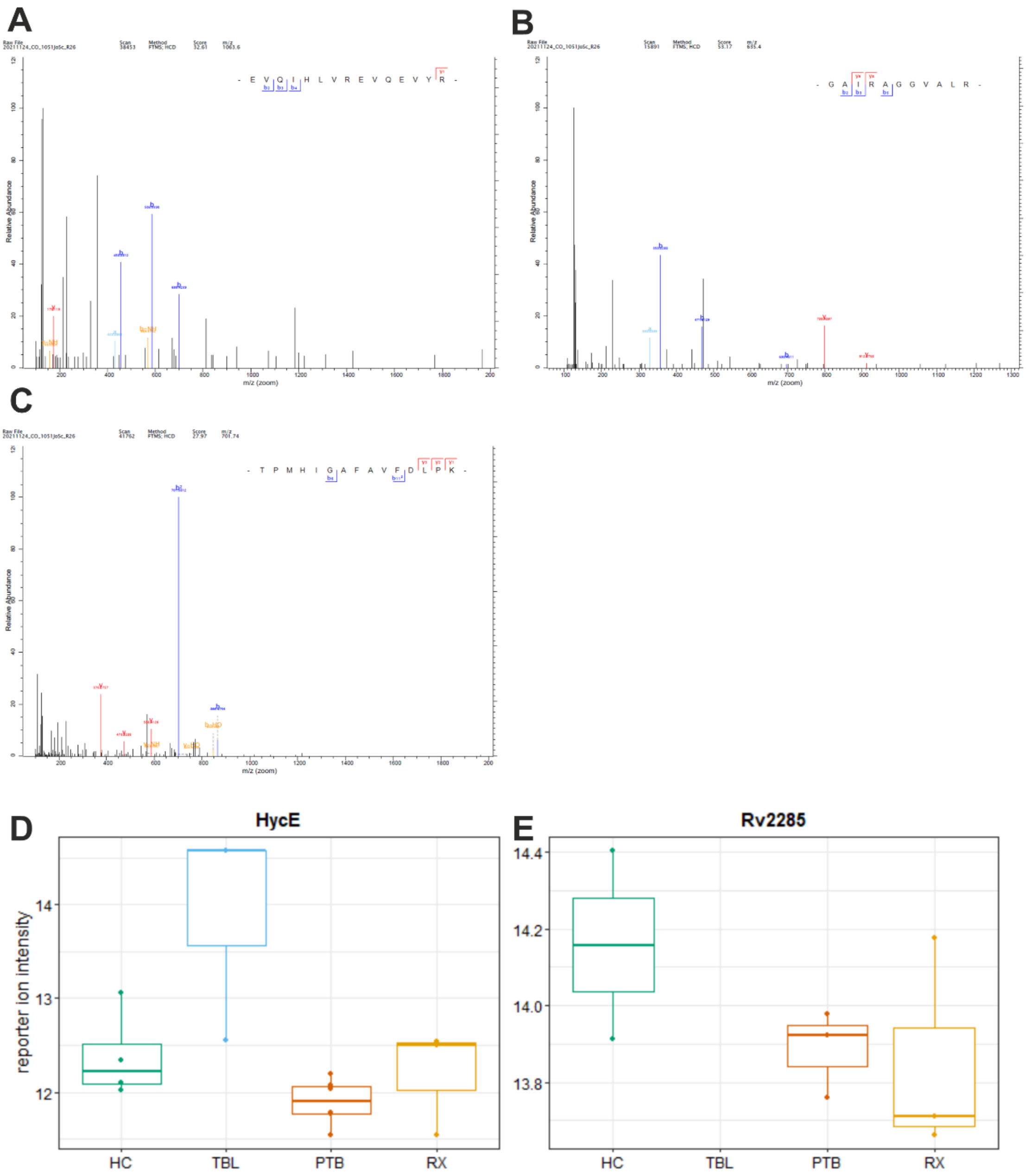


**Figure S5. Identification and quantification of *M. tuberculosis* proteins in circulating exosomes.** RopC, HycE and Rv2285 of *Mtb* origin were identified in circulating exosomes. Annotated mass spectra were extracted from MaxQuant viewer (**A-C**). Comparative quantification was performed by normalized TMT reporter ion intensities (**D** and **E**). HC - healthy controls, PTB - pulmonary tuberculosis, TBL – tuberculous lymphadenitis, and Rx - PTB after anti-TB drug treatment.

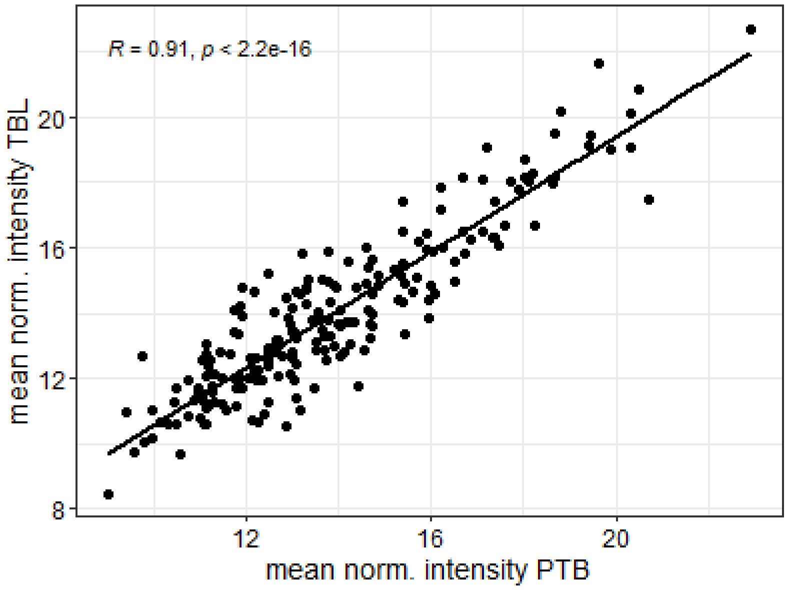


**Figure S6. Correlation of PTB and TBL samples.** Mean values of normalized intensities of PTB-derived (x-axis) and TBL-derived samples (y-axis) are correlated. PTB - pulmonary tuberculosis and TBL – tuberculous lymphadenitis..
