# Supplementary material for: Mycobacterium tuberculosis Affects Protein and Lipid Content of Circulating Exosomes in Infected Patients Depending on Tuberculosis Disease State
Source: Biomedicines. 2022 Mar 27;10(4):783. doi: 10.3390/biomedicines10040783 (PMC9025801; doi:10.3390/biomedicines10040783)
Supplement: Supplementary file 1 [file biomedicines-10-00783-s001.zip › Table S1.pdf]

Table S1. Sociodemographic characteristics of study participants*. Overview on sociodemographic parameters of study individuals. Numbers indicate absolute values of individuals.

	HC	PTB	TBL	Rx
**Sex**				
**Male**	9	12	14	6
**Female**	1	7	5	3
**Age in years**				
**0-24**	2	6	6	4
**25-44**	6	9	8	5
**≥45**	2	4	5	0
**Residence**				
**Urban**	1	5	8	8
**Rural**	9	14	11	1
**Education**				
**Illiterate**	3	8	6	1
**Above read and Write**	6	11	13	8
**Smoking**				
**Yes**	0	0	0	0
**No**	10	19	19	9
**Alcohol intake**				
**Yes**	5	13	11	4
**No**	5	6	8	5
**Chatting**				
**Yes**	0	1	0	0
**No**	10	18	19	9
**Comorbidity**				
**Yes**	0	0	0	0
**No**	10	19	19	9
**TB history**				
**Yes, have taken and completed anti- TB treatment**	0	0	0	9
**No**	10	19	19	0

*Because of the small number of exosomes, 1 HC, 6PTB, 6 TBL, and 3Rx samples were excluded from exosome lipid analysis. HC- healthy control, PTB- pulmonary tuberculosis, TBL-tuberculosis lymphadenitis and Rx- pulmonary tuberculosis cases after anti-TB treatment
